# Brain potentials evoked by intraepidermal electrical stimuli reflect the central sensitization of nociceptive pathways

**DOI:** 10.1152/jn.00013.2016

**Published:** 2016-04-20

**Authors:** M. Liang, M. C. Lee, J. O'Neill, A. H. Dickenson, G. D. Iannetti

**Affiliations:** ^1^School of Medical Imaging, Tianjin Medical University, Tianjin, China;; ^2^Department of Neuroscience, Physiology and Pharmacology, University College London, London, United Kingdom; and; ^3^Division of Anaesthesia, University of Cambridge, Cambridge, United Kingdom

**Keywords:** central sensitization, secondary hyperalgesia, mechanical punctate stimulation, intraepidermal electrical stimulation, EEG

## Abstract

Central sensitization (CS), the increased sensitivity of the central nervous system to somatosensory inputs, accounts for secondary hyperalgesia, a typical sign of several painful clinical conditions. Brain potentials elicited by mechanical punctate stimulation using flat-tip probes can provide neural correlates of CS, but their signal-to-noise ratio is limited by poor synchronization of the afferent nociceptive input. Additionally, mechanical punctate stimulation does not activate nociceptors exclusively. In contrast, low-intensity intraepidermal electrical stimulation (IES) allows selective activation of type II Aδ-mechano-heat nociceptors (II-AMHs) and elicits reproducible brain potentials. However, it is unclear whether hyperalgesia from IES occurs and coexists with secondary mechanical punctate hyperalgesia, and whether the magnitude of the electroencephalographic (EEG) responses evoked by IES within the hyperalgesic area is increased. To address these questions, we explored the modulation of the psychophysical and EEG responses to IES by intraepidermal injection of capsaicin in healthy human subjects. We obtained three main results. First, the intensity of the sensation elicited by IES was significantly increased in participants who developed robust mechanical punctate hyperalgesia after capsaicin injection (i.e., responders), indicating that hyperalgesia from IES coexists with punctate mechanical hyperalgesia. Second, the N2 peak magnitude of the EEG responses elicited by IES was significantly increased after the intraepidermal injection of capsaicin in responders only. Third, a receiver-operator characteristics analysis showed that the N2 peak amplitude is clearly predictive of the presence of CS. These findings suggest that the EEG responses elicited by IES reflect secondary hyperalgesia and therefore represent an objective correlate of CS.

## Abstract (precis)

*Secondary mechanical punctate hyperalgesia is a cardinal sign of central sensitization (CS), an important mechanism of chronic pain. Our study demonstrates that hyperalgesia from intraepidermal electrical stimulation coexists with mechanical punctate hyperalgesia and elicits electroencephalographic (EEG) potentials that predict the occurrence of punctate hyperalgesia in a human experimental model of CS. These findings inform clinical development of EEG-based biomarkers of CS*.

## NEW & NOTEWORTHY

*Secondary mechanical punctate hyperalgesia is a cardinal sign of central sensitization (CS), an important mechanism of chronic pain. Our study demonstrates that hyperalgesia from intraepidermal electrical stimulation coexists with mechanical punctate hyperalgesia and elicits electroencephalographic (EEG) potentials that predict the occurrence of punctate hyperalgesia in a human experimental model of CS. These findings inform clinical development of EEG-based biomarkers of CS*.

central sensitization (CS) refers to the increased sensitivity of the central nervous system to somatosensory inputs. CS accounts for the enhanced painful percepts elicited by nociceptive stimulation of the skin surrounding a site of tissue injury (secondary hyperalgesia; Ringkamp et al. 2013), and it has been suggested to be an important contributor to several chronic pain states (Ji et al. 2003; Latremoliere and Woolf 2009). A cardinal sign of CS is secondary hyperalgesia to nociceptive punctate mechanical stimuli, also known as secondary mechanical punctate hyperalgesia. Punctate stimuli, when delivered using flat-tip probes, preferentially activate the free nerve endings of type I Aδ-mechano-heat nociceptors (I-AMH; Magerl et al. 2001). CS is typically established by an intense activation of C-fiber skin nociceptors: the resulting afferent barrage to the dorsal horn results in a heterosynaptic facilitation of I-AMH inputs, which substantiates secondary mechanical punctate hyperalgesia (Geber et al. 2007; Ziegler et al. 1999).

Secondary mechanical punctate hyperalgesia has been quantified by measuring the brain activity using noninvasive functional neuroimaging techniques, such as functional magnetic resonance imaging (fMRI; Lee et al. 2008) and magnetoencephalography (MEG; Maihofner et al. 2010). Given that secondary hyperalgesia is a well-established surrogate model for centrally generated hyperalgesia in chronic pain patients, such neural correlates have potential clinical and pharmaceutical applications. However, fMRI and MEG are costly and not readily available. In contrast, electroencephalography (EEG) is more affordable and routinely used in clinical practice. Moreover, previous studies have shown that punctate stimulation causing pinprick-like pain can elicit EEG potentials whose amplitudes reflect subjective reports of secondary mechanical punctate hyperalgesia (Davies et al. 2010; Iannetti et al. 2013). However, there are technical and physiological constrains that may hamper clinical translation of pinprick-evoked potentials. First, the mechanical stimulus is generated by hand-held probes. The use of hand-held probes is operator dependent, which limits reproducibility of stimulus delivery. Second, given that the force exerted is driven passively by a weighted cylinder (Magerl et al. 2001), the probe needs to be held perpendicularly to both the skin and the ground to ensure that a consistent force is applied. This limits the number of body territories that can be effectively stimulated. Pneumatically driven (Kohlloffel et al. 1991) or solenoid-powered (Davies et al. 2010) mechanical devices also have been described: they circumvent some of the difficulties associated with the use of hand-held probes. However, any device that relies on mechanical stimulation to activate cutaneous nociceptors remains limited by a crucial factor, the variability in skin compliance. This limits the synchronicity of nociceptor activation, introduces high variability of spatial and temporal summation at central synapses, and thus makes the estimation of response latency and amplitude difficult. Third, the spatial location of the mechanically stimulated spot is typically changed between trials, which further increases the variability of the afferent nociceptive input. Last, and most importantly, mechanical punctate stimulation activates intraepidermal nociceptive nerve endings preferentially, but not selectively. Indeed, at higher stimulus intensities the dermis and subcutaneous tissues are more likely to become temporarily deformed, which may result in a certain degree of activation of deeper Aβ-afferents (Treede et al. 2002).

A possible alternative to punctate stimulation is the selective activation of Aδ-nociceptors by simple and affordable concentric electrodes that are designed to deliver currents exclusively to the epidermal skin layers, where the free nerve endings of nociceptors ramify (Inui and Kakigi 2012; Inui et al. 2002). Psychophysical, behavioral, and electrophysiological data indicate that when used at low intensity of current, intraepidermal electrical stimulation (IES) activates Aδ-nociceptors selectively, i.e., without coactivating Aβ-afferents (Mouraux et al. 2010). Still, it remains to be determined whether the psychophysical and EEG responses evoked by IES are increased in the presence of secondary mechanical punctate hyperalgesia. This question is physiologically pertinent: given the evidence that IES predominantly activate type II AMHs (Mouraux et al. 2010; Treede and Magerl 2000), the observation that EEG responses to IES are increased would imply that hyperalgesia from IES is also mediated by this class of nociceptive afferents.

In the present study, we explored whether IES-evoked potentials hold promise as an objective neural correlate of secondary hyperalgesia. We intraepidermally injected capsaicin in the right hand of healthy subjects to induce a state of CS. Participants were classified as responders or nonresponders on the basis of whether or not they developed robust secondary mechanical punctate hyperalgesia. We then *1*) tested whether subjects who developed secondary mechanical hyperalgesia also developed secondary hyperalgesia from nociceptive-specific IES, *2*) explored whether the magnitude of the EEG responses to nociceptive IES delivered to the secondary hyperalgesic area was significantly increased, and *3*) quantified the sensitivity and specificity of the EEG responses elicited by IES for detecting the presence of secondary hyperalgesia in our study cohort.

## MATERIALS AND METHODS

### Participants

Fourteen healthy right-handed volunteers participated in this study. All participants were pain-free, not taking any medication, and had no history of severe allergic reactions to chili peppers at the time of testing. They all gave signed written informed consent, and the experimental procedures were approved by the UCL Research Ethics Committee. Before the electrophysiological recording, the experimental setup and the psychophysical rating task were clearly explained to the participants, who were also familiarized with the sensation elicited by IES. Data from two participants were discarded because no clear event-related potential (ERP) could be identified, and the data from the remaining 12 participants (age 22–39 yr, 7 female) were analyzed.

### Experimental Design

**Fig. 1.**
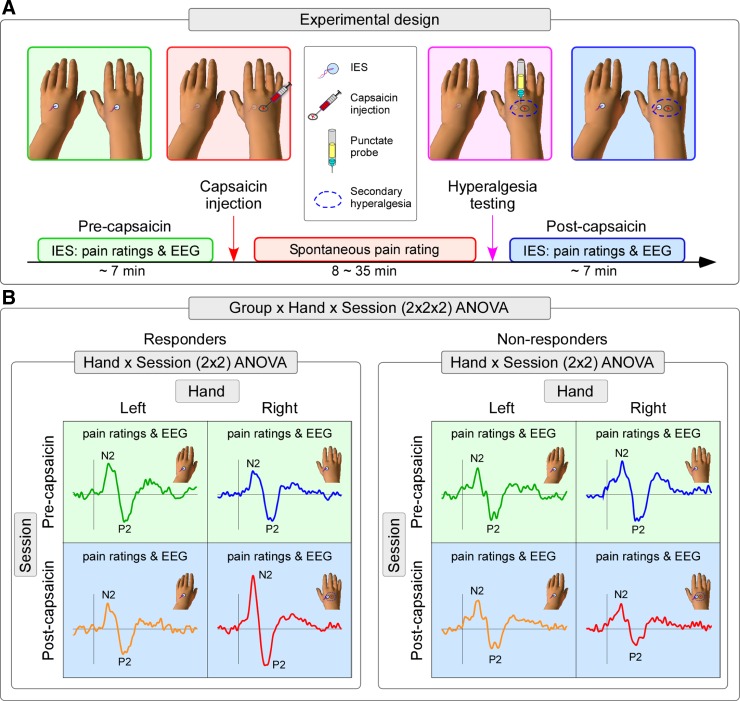
*A*: experimental design. The state of central sensitization was induced by intraepidermal injection of capsaicin (red arrow on the timeline). Capsaicin-induced spontaneous pain lasted between 8 and 35 min, during which pain ratings were collected every 10 s during the first 3 min and then every 30 s until the pain intensity ratings were less than 5 out of 100 (red box). Psychophysical and EEG responses to intraepidermal electrical stimulation (IES) were collected before capsaicin injection (i.e., pre-capsaicin session; green box) and after capsaicin-induced spontaneous pain had disappeared (i.e., post-capsaicin session; blue box). The development of secondary hyperalgesia to punctate mechanical stimuli was assessed by the ratio of the subjective intensity ratings of the sensation evoked by stimulation of the right and the left hand (Right/Left; purple arrow on the timeline). Participants were considered responders if the ratio was ≥2, and nonresponders otherwise. *B*: schematic of the statistical analysis. A 3-way ANOVA with the factors of Group (responders, nonresponders), Session (pre-capsaicin, post-capsaicin) and Hand (left, right) was used to analyze both psychophysical and event-related potential (ERP) responses. The three-way interaction (Group × Session × Hand) indicated the effect of central sensitization on these responses. Further post hoc 2-way ANOVAs with the factors of Session and Hand were performed to define the effect within each group.

The experimental design is summarized in Fig. 1*A*. Experiments were conducted in a silent and temperature-controlled room. Throughout the experiment participants sat on a comfortable chair with their hands resting on a table in front of them. Participants were instructed to keep their gaze fixed on a black cross (2 × 2 cm) placed centrally in front of them, at a distance of 1.5 m, ∼20° below eye level. To induce CS, capsaicin was injected intraepidermally on the right hand dorsum (Ziegler et al. 1999). IES were delivered in two separate blocks, one before (“pre-capsaicin”) and one after capsaicin injection (“post-capsaicin”). In the post-capsaicin block, IES were delivered only after capsaicin-induced spontaneous pain had resolved. In each block we delivered 20 stimuli on the left hand dorsum and 20 stimuli on the right hand dorsum, in pseudorandom order, with an interstimulus interval (ISI) of 8–12 s (rectangular distribution). Therefore, there were four conditions: *1*) pre-capsaicin, right hand (PreRH); *2*) pre-capsaicin, left hand (PreLH); *3*) post-capsaicin, right-hand (PostRH); and *4*) post-capsaicin, left hand (PostLH). Three seconds after the stimulus onset, subjects were asked to state whether the stimulus was delivered on the right or the left hand and to provide ratings of the perceived intensity of pinprick pain using a numerical scale ranging from 0 (no pinprick sensation) to 100 (the most intense pinprick sensation imaginable).

### Intraepidermal Electrical Stimulation

IES consisted of two constant-current square-wave pulses delivered in rapid succession, as described previously (Inui et al. 2002; Mouraux et al. 2010). Each pulse lasted 500 μs, and the interpulse interval was 10 ms (DS7; Digitimer, Welwyn Garden City, UK). Stimuli were delivered using a stainless steel concentric bipolar needle electrode consisting of a needle cathode (length, 0.1 mm; diameter, 0.2 mm) surrounded by a cylindrical anode (diameter, 1.4 mm) (Inui et al. 2002; Mouraux et al. 2010). By gently pressing the device against the skin, the needle electrode was inserted into the epidermis. Two electrodes were applied, one on the dorsum of each hand. Once the electrodes were fixed, the thresholds for stimulus perception were determined for each hand and each subject using an adaptive staircase procedure. The final intensity of the IES for the experiment was set to twice the perceptual threshold to ensure selective stimulation of skin nociceptors (Mouraux et al. 2010).

After the thresholding procedure, we delivered a few stimuli at the intensity determined above, to familiarize the participant with the elicited sensation. The locations of the electrodes were adjusted on each participant until the reported intensities on both hands were similar, and then the thresholding procedure was repeated and the new stimulus intensity determined.

### Capsaicin Injection

To induce CS, we injected intraepidermally a 10 mM solution of capsaicin (40 μg in a 12.5-μl volume of normal saline containing 0.16% Tween 80; for details, see LaMotte et al. 1991). The capsaicin solution was injected at an angle of ∼15° to the skin surface using a 27-gauge disposable needle. The injection site was ∼1.5 cm away from the IES electrode on the right hand dorsum. Therefore, IES was delivered on the skin area of secondary hyperalgesia away from the injection site where the skin would have been numbed by the local neurotoxic effects of capsaicin (LaMotte et al. 1992).

### Capsaicin-Induced Spontaneous Pain and Secondary Hyperalgesia Assessment

Spontaneous pain intensity after capsaicin injection was recorded using a numerical rating scale ranging between 0 (no pain) and 100 (worst pain imaginable). Participants were required to rate verbally the intensity of spontaneous pain every 10 s during the first 3 min and then every 30 s until the pain intensity ratings were less than 5 out of 100.

**Fig. 2.**
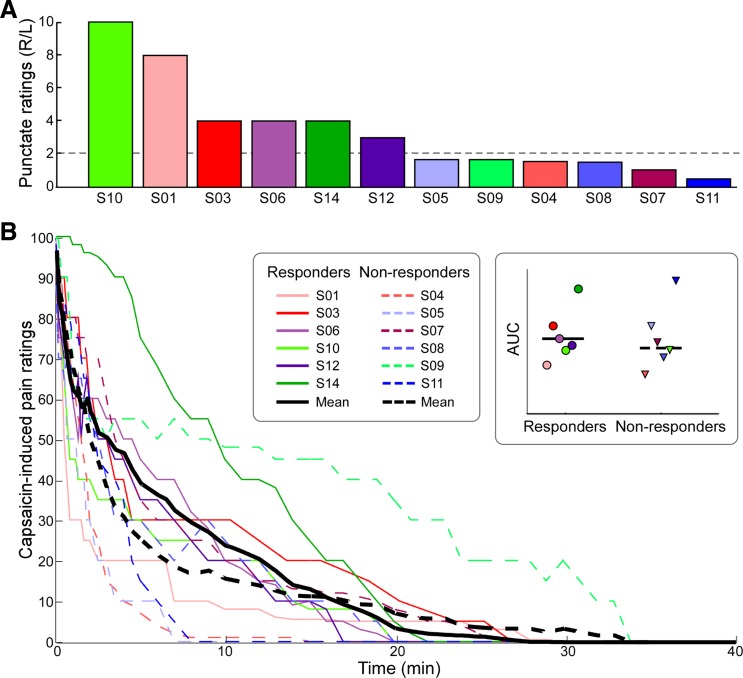
*A*: participants were divided into 2 groups according to the ratio of ratings to punctate stimulation of the right (R) and left (L) hands: participants who rated the intensity of right hand stimulation as at least twice that of the left hand stimulation were classified as responders. Participants were sorted by the ratio of reported intensity ratings (R/L), in descending order. *B*: time course of capsaicin-induced pain ratings. Single participants are color coded. Solid lines indicate responders. All participants rated the pain intensity between 90 and 100 at the moment of the injection. Pain ratings decreased fairly quickly over time. *Inset*: comparison of the mean area under the curve (AUC) between responders and nonresponders revealed no significant difference (*t*_10_ = 0.39, *P* = 0.70). Colored symbols indicate single-subject AUC data.

The development of mechanical hyperalgesia in the skin area surrounding the injection site was confirmed by punctate mechanical stimulation of the skin adjacent (within 1 cm) to the external circumference of the concentric IES electrode using a flat-tip punctate probe (256 mN). This probe comprises a stainless steel wire tip (diameter, 0.25 mm) attached to a mounted weight (256 mN) that glides smoothly within a hollow handheld cylindrical tube. When the probe is applied perpendicularly to the skin, its weight rests entirely on the wire tip, thus exerting a constant force of 256 mN. More details and a depiction of the punctate probe can be found in a previous report (Iannetti et al. 2013), as well as on the manufacturer website (MRC Systems; http://www.mrc-systems.de/en/products/pinprick). The same mechanical stimulus was applied to the corresponding position of the left hand, to obtain a baseline for quantifying the effect of secondary hyperalgesia, as follows. Participants were asked to report the intensity of punctate stimulation of the right hand (capsaicin injected) and of the left hand (control) using a numerical rating scale that ranged between 0 (no pinprick sensation) and 100 (the most intense pinprick sensation imaginable). For each hand, punctate stimuli were applied three times, with an ISI of ∼5 s, after the spontaneous pain induced by the capsaicin injection in the right hand had decreased to less than 5 out of 100 (Fig. 2). For every individual, the mean ratings of the sensations elicited by the three stimuli was obtained for each hand and each condition. The intensity of secondary hyperalgesia was quantified as the ratio of the subjective ratings of the pinprick sensation elicited by mechanical stimulation of the right and the left hands (Right/Left). Participants were considered to have developed robust secondary hyperalgesia from punctate stimuli if the ratio was ≥2 and were thus classified as responders. All other participants were classified as nonresponders. This ratio was chosen on the basis of a previous EEG study, which showed that an approximately twofold increase (+93%) in pinprick sensation elicited by punctate stimulation after capsaicin sensitization was associated with significant increases in the evoked EEG response (Iannetti et al. 2013).

### EEG Recording

The EEG was recorded using 31 Ag-AgCl electrodes placed on the scalp according to the International 10-20 system and referenced to the nose. Ocular movements and eye blinks were recorded using two surface electrodes, one placed over the right lower eyelid and the other placed ∼1 cm lateral to the lateral corner of the right orbit. Signals were amplified and digitized using a sampling rate of 1,024 Hz (SD32; Micromed, Mogliano Veneto, Italy).

### Behavioral Data Analysis

Single-trial ratings of the sensation elicited by IES were first normalized between 0 and 100 for each participant (the minimum value was set to 0 and the maximum value was set to 100). This procedure mitigates the differences in the range of values on the numerical rating scale with which individuals reported the intensity of pinprick pain elicited by IES (Huang et al. 2013). Normalized stimulus intensity ratings were subsequently averaged across trials for each condition, resulting in four average values for each participant (PreRH, PreLH, PostRH, and PostLH).

To test whether capsaicin injection had an effect on the perceived IES intensity, we performed a three-way ANOVA with the following experimental factors: Group (2 levels: responders, nonresponders), Session (2 levels: pre-capsaicin, post-capsaicin), and Hand [2 levels: injected (right), control (left)]. Where effects were significant, post hoc analyses were performed to define their direction and possible interactions. Two-way repeated-measures ANOVA for the main and interaction effects of Session and Hand were performed to define the effects of capsaicin injection on the intensity of the sensation elicited by IES within each group. The statistical threshold of the post hoc analyses was determined by Bonferroni correction accounting for the number of comparisons (*P* = 0.05/2 = 0.025).

### EEG Data Analyses

EEG data analyses were performed using Letswave (www.nocions.org; Mouraux and Iannetti 2008) and MATLAB (The MathWorks, Natick, MA). Continuous EEG recordings were segmented into epochs using a time window of 2 s (−0.5 to 1.5 s relative to the stimulus onset). Each epoch was baseline corrected (baseline interval ranging from −0.2 to 0 s) and bandpass filtered (1–30 Hz). Artifacts produced by eye blinks or eye movements were subtracted using a validated method based on independent component analysis (Jung et al. 2000). In all data sets, independent components related to eye movements had a large electrooculogram channel contribution and a frontal scalp distribution. In addition, epochs with amplitude values exceeding ±100 μV were rejected from further analysis. These epochs constituted 0.6 ± 1.8% (mean ± SD across all conditions and participants) of the total number of epochs. Remaining epochs were then averaged for each condition, resulting in four average ERP waveforms for each participant.

The N2-P2 complex was measured at the vertex (Cz), and it was defined as the largest negative-positive deflection occurring after stimulus onset. The amplitude of both the N2 and P2 peaks were calculated for each condition and participant and then tested for the effect of capsaicin injection using the same three-way ANOVA described for the behavioral data (Fig. 1*B*). Because two peaks (N2 and P2) were tested, the statistical threshold was determined by Bonferroni correction accounting for the number of peaks (*P* = 0.05/2 peaks = 0.025). Where effects were significant, the same post hoc analyses described for the behavioral data (i.e., 2-way repeated-measures ANOVA) were performed for each group, and the same statistical threshold, Bonferroni corrected (*P* = 0.05/2 groups = 0.025), was used to determine the significance of the post hoc results. The latency of the N2 and P2 peaks was analyzed using the same procedure.

To test the predictive value of ERP amplitude for the presence of CS, we plotted the receiver operating characteristic (ROC) curves obtained using the interaction term [i.e., (PostRH − PreRH) − (PostLH − PreLH)] calculated for the N2-wave and P2-wave peak amplitudes. The true positive rate (sensitivity) is plotted against the false positive rate (100 − specificity) for different cutoff values of the interaction terms. Each point on the ROC curve represents a sensitivity/specificity pair corresponding to a particular decision threshold for the interaction term. Above each of these thresholds, the individual is predicted to be a responder, and vice versa. If interaction terms had perfect classification performance, their ROC curves would pass through the upper left corner (100% sensitivity, 100% specificity). The closer the ROC curve is to the upper left corner, the higher the overall accuracy of the interaction term is in distinguishing responders and nonresponders (Zweig and Campbell 1993). The area under the curve (AUC) is typically used to quantify the classification performance. An AUC value of 0.5 corresponds to a random classification (i.e., to a useless test), whereas an AUC of 1.0 indicates that the test performs perfectly. We calculated the AUC for the interaction terms obtained from the amplitude of the N2 and P2 peaks to assess their sensitivity and specificity for detecting the presence of a CS state. We tested whether the AUC size of each measure was significantly greater than 0.5 (Hanley and McNeil 1982).

## RESULTS

### Capsaicin-Induced Spontaneous Pain

Six of 12 participants developed robust secondary hyperalgesia on the capsaicin-treated hand and were therefore classified as responders (Fig. 2*A*). The time courses of the capsaicin-induced pain for all subjects are shown in Fig. 2*B*. In the first few seconds after the injection, capsaicin induced a very intense sensation of burning pain, which decreased exponentially over time (Lee et al. 2008; Magerl et al. 1998). The time course of spontaneous pain ratings for each subject was summarized as AUC. The AUC values for responders and nonresponders were compared using a two-sample *t*-test. The result showed no significant difference in capsaicin-induced spontaneous pain between the two groups (*t*_10_ = 0.39, *P* = 0.70; Fig. 2*B*, *inset*). This observation suggests that both groups perceived the conditioning stimulus (i.e., the intraepidermal injection of capsaicin) similarly.

### Psychophysics of Intraepidermal Stimulation of the Area of Secondary Mechanical Punctate Hyperalgesia

**Fig. 3.**
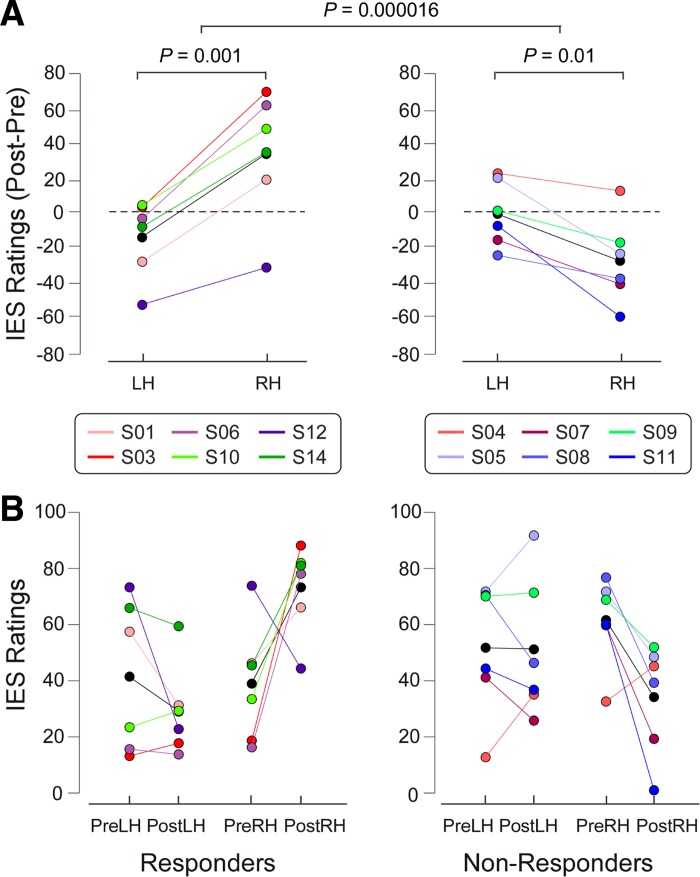
Subjective intensity ratings of the sensation elicited by the IES of responders (*left*) and nonresponders (*right*). *A*: to highlight the interaction between the factors Session and Hand, the subtracted ratings (post- minus pre-capsaicin injection) are shown for each hand. Colored circles indicate single subjects, and black circles indicate the group average for each condition. Two-way ANOVA revealed that responders had a highly significant interaction between the factors Session and Hand. This reveals a capsaicin-induced increase of IES ratings (Post − Pre) on the right hand. In contrast, in nonresponders the 2-way ANOVA revealed a decrease of IES ratings on the right hand compared with those on the left hand. These differences in the capsaicin effect on IES ratings between responders and nonresponders were confirmed by the 3-way ANOVA, which revealed a highly significant triple interaction (Group × Session × Hand; comparison between *left* and *right* panels). LH, left hand; RH, right hand. *B*: individual values (colored circles) and mean value (black circles) for each condition. PreLH, pre-capsaicin, left hand; PostLH: post-capsaicin, left hand; PreRH: pre-capsaicin, right hand; PostRH: post-capsaicin, right hand.

All subjects correctly reported whether each IES was delivered to the right or the left hand in all trials. The three-way ANOVA on the subjective ratings of perceived IES intensity showed a two-way interaction between Group and Hand (*F*_1,10_ = 9.02, *P* = 0.01) and, more importantly, a clear three-way interaction between Group, Session, and Hand (*F*_1,10_ = 59.27, *P* = 0.000016; Fig. 3). No other significant effects were detected (Table 1). This finding indicates that responders perceived right-hand stimulation as more painful than left-hand stimulation but only after capsaicin was injected in the right hand. The results of all post hoc two-way ANOVAs are shown in Table 2. Both responders (*F*_1,5_ = 49.79, *P* = 0.001) and nonresponders (*F*_1,5_ = 15.19, *P* = 0.01) showed significant interactions between Session and Hand, but in opposite directions: the responders had clearly increased ratings on their treated hand after capsaicin injection, whereas the nonresponders showed mildly decreased ratings on their treated hand after capsaicin injection (Fig. 3). The results demonstrate a clear secondary hyperalgesia from both IES and mechanical punctate stimulation after capsaicin injection.

**Table 1.** Results of 3-way ANOVA of psychophysical and EEG responses elicited by IES

		ERP Peak Amplitude		ERP Peak Latency	
3-Way ANOVA	Pain Intensity Ratings	N2	P2	N2	P2
Main effect of Group	*F*_1,10_ = 0.20	*F*_1,10_ = 0.08	*F*_1,10_ = 0.15	*F*_1,10_ = 0.008	*F*_1,10_ = 1.51
	*P* = 0.665	*P* = 0.778	*P* = 0.705	*P* = 0.930	*P* = 0.247
Main effect of Session	*F*_1,10_ = 0.05	*F*_1,10_ = 0.268	*F*_1,10_ = 2.65	*F*_1,10_ = 0.50	*F*_1,10_ = 0.38
	*P* = 0.833	*P* = 0.61	*P* = 0.134	*P* = 0.498	*P* = 0.553
Main effect of Hand	*F*_1,10_ = 4.52	*F*_1,10_ = 1.60	*F*_1,10_ = 0.52	*F*_1,10_ = 7.41	*F*_1,10_ = 0.11
	*P* = 0.059	*P* = 0.234	*P* = 0.487	***P* = 0.022**	*P* = 0.742
2-way interaction: Group × Session	*F*_1,10_ = 3.04	*F*_1,10_ = 1.40	*F*_1,10_ = 11.13	*F*_1,10_ = 0.05	*F*_1,10_ = 0.53
	*P* = 0.112	*P* = 0.265	***P* = 0.008**	*P* = 0.827	*P* = 0.484
2-way interaction: Group × Hand	*F*_1,10_ = 9.02	*F*_1,10_ = 0.45	*F*_1,10_ = 1.42	*F*_1,10_ = 0.11	*F*_1,10_ = 0.0003
	***P* = 0.013**	*P* = 0.517	*P* = 0.261	*P* = 0.751	*P* = 0.987
2-way interaction: Session × Hand	*F*_1,10_ = 4.31	*F*_1,10_ = 0.19	*F*_1,10_ = 1.27	*F*_1,10_ = 4.33	*F*_1,10_ = 1.19
	*P* = 0.065	*P* = 0.674	*P* = 0.286	*P* = 0.064	*P* = 0.301
3-way interaction: Group × Session × Hand	*F*_1,10_ = 59.27	*F*_1,10_ = 7.84	*F*_1,10_ = 2.04	*F*_1,10_ = 0.37	*F*_1,10_ = 0.06
	***P* = 0.000016**	***P* = 0.019**	*P* = 0.184	*P* = 0.559	*P* = 0.813

Data are *F* statistics and *P* values from 3-way ANOVA with factors Group, Session, and Hand. Significant effects are highlighted in bold.

**Table 2.** Psychophysical and EEG responses elicited by IES for each condition and results of post hoc 2-way ANOVA for each group

	Condition						
Group	PreRH	PostRH	PreLH	PostLH	Main Effect of Session (Pre vs. Post)	Main Effect of Hand (LH vs. RH)	Interaction (Session × Hand)
*Pain intensity ratings*							
Responders	39.0 ± 21.3	73.3 ± 15.9	41.5 ± 27.1	29.0 ± 16.3	*F*_1,5_ = 0.87 *P* = 0.394	***F*_1,5_ = 11.59 *P* = 0.019**	***F_1,5_* = 49.79 *P* = 0.0009**
Nonresponders	61.5 ± 15.7	34.1 ± 20.0	51.6 ± 23.6	51.1 ± 25.3	*F*_1,5_ = 2.90 *P* = 0.150	*F*_1,5_ = 0.44 *P* = 0.535	***F***_*1,5*_** = 15.19 *P* = 0.011**
*N2 peak amplitudes, μV*							
Responders	−8.6 ± 4.4	−14.0 ± 6.4	−9.9 ± 5.4	−8.9 ± 5.1	*F*_1,5_ = 1.66 *P* = 0.254	*F*_1,5_ = 2.55 *P* = 0.171	***F***_*1,5*_** = 15.15 *P* = 0.011**
Nonresponders	−12.9 ± 5.5	−9.7 ± 5.1	−10.0 ± 6.2	−11.5 ± 2.1	*F*_1,5_ = 0.20 *P* = 0.676	*F*_*1,5*_ = 0.14 *P* = 0.723	*F*_*1,5*_ = 1.69 *P* = 0.250
*P2 peak amplitudes, μV*							
Responders	7.9 ± 2.3	10.6 ± 2.5	8.3 ± 3.5	7.6 ± 1.4	*F*_1,5_ = 4.32 *P* = 0.092	*F*_1,5_ = 1.87 *P* = 0.230	*F*_*1,5*_ = 9.77 *P* = 0.026
Nonresponders	10.7 ± 3.6	7.4 ± 4.2	10.8 ± 3.2	7.9 ± 3.8	*F*_1,5_ = 7.42 *P* = 0.042	*F*_*1,5*_ = 0.11 *P* = 0.755	*F*_*1,5*_ = 0.03 *P* = 0.875
*N2 peak latency, ms*							
Responders	165 ± 29	136 ± 16	168 ± 29	178 ± 47	*F*_1,5_ = 0.68 *P* = 0.447	*F*_*1,5*_ = 8.75 *P* = 0.032	*F*_*1,5*_ = 5.94 *P* = 0.059
Nonresponders	162 ± 38	147 ± 47	169 ± 44	175 ± 39	*F*_1,5_ = 0.08 *P* = 0.783	*F*_*1,5*_ = 1.95 *P* = 0.221	*F*_*1,5*_ = 0.78 *P* = 0.417

Data are normalized pain intensity ratings, peak amplitudes, and peak latency in each condition, as well as *F* statistics and *P* values from post hoc 2-way ANOVA with factors Session and Hand, for each group. Significant effects are highlighted in bold.

### ERP Waveforms

**Fig. 4.**
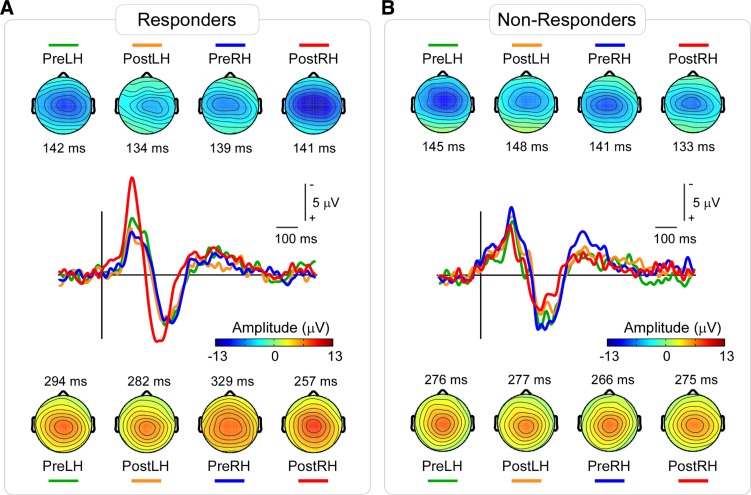
Group-average ERP waveforms and scalp maps elicited by IES in responders (*A*) and nonresponders (*B*). Waveforms at the channel Cz in different conditions are shown in different colors. The ERP elicited by IES stimuli clearly increased after capsaicin injection only on the right hand in responders. Scalp maps at the N2 peak latencies show a central distribution, slightly lateralized to the hemisphere contralateral to the stimulated hand, maximal at the vertex (*top*). Scalp maps at the P2 peak latencies show a central distribution, maximal at the vertex (*bottom*). Color bar shows the ERP amplitude in scalp maps.

ERPs elicited by IES stimuli showed a clear N2–P2 complex maximal at electrode Cz in all four conditions of each group. Grand-average waveforms and scalp maps at N2 and P2 peak latencies are shown in Fig. 4. The ERP amplitude increased after capsaicin injection in the right hand of the responders compared with all other conditions. Statistical comparisons of peak amplitude and latency of the N2 and P2 waves across different conditions and groups are reported below and summarized in Tables 1 and 2.

#### N2 peak amplitude.

**Fig. 5.**
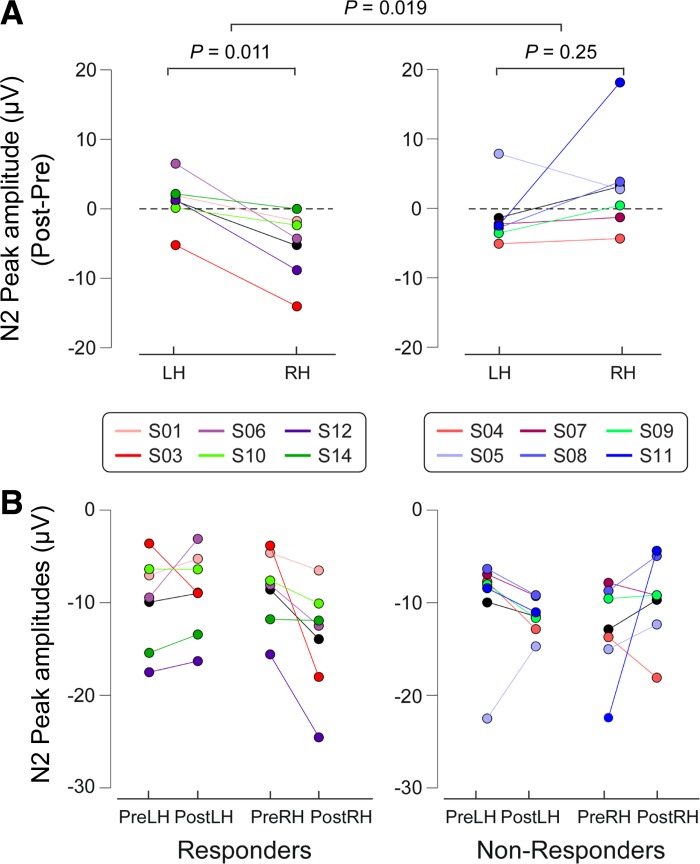
ERP amplitudes (N2) of IES of responders (*left*) and nonresponders (*right*). *A*: to highlight the interaction between Session and Hand in each group, the subtracted ERP amplitudes (post- minus pre-capsaicin injection) are shown for each hand. Colored circles indicate single subjects, and black circles indicate the group average for each condition. Two-way ANOVA revealed that responders had a significant interaction between the factors Session and Hand. This reveals a capsaicin-induced increase of IES ERP amplitudes (Post-Pre) on the right hand. In contrast, in nonresponders the 2-way ANOVA did not show any significant effect. These differences in the capsaicin effect on ERP amplitudes between responders and nonresponders were confirmed by the 3-way ANOVA, which revealed a significant triple interaction (Group × Session × Hand; comparison between *left* and *right* panels). *B*: individual values (colored circles) and mean value (black circles) for each condition.

The three-way ANOVA of N2 peak amplitudes showed a three-way interaction between Group, Session, and Hand (*F*_1,10_ = 7.84, *P* = 0.019). No other significant effects were detected (Table 1). Hence, N2 peak amplitudes at Cz were greater following right-hand IES compared with left-hand IES in the responders, but only when IES were delivered to the hand where capsaicin had been injected (i.e., the right hand). Post hoc two-way ANOVAs (Table 2) revealed that only responders showed an interaction between Session and Hand (*F*_1,5_ = 15.15, *P* = 0.011), indicating increased N2 amplitudes on their treated hand after capsaicin injection. Figure 5 shows the single subjects' N2 peak amplitudes, as well as the statistical results.

#### P2 peak amplitude.

The three-way ANOVA of P2 peak amplitudes showed that there was a two-way interaction between Group and Session (*F*_1,10_ = 11.13, *P* = 0.008). This effect was caused by an overall increased P2 amplitude in the post-capsaicin session of responders but a decreased P2 amplitude in the post-capsaicin session of nonresponders. No other significant effects were detected (Table 1). Post hoc two-way ANOVAs (Table 2) showed that there was a trend for an interaction between Session and Hand that, however, did not survive correction for multiple comparisons in responders (*F*_1,5_ = 9.77, *P* = 0.026): in this group, P2 amplitudes in the post-capsaicin session, compared with those in the pre-capsaicin session, were increased following right-hand stimulation and slightly decreased following left-hand stimulation.

#### N2 peak latency.

The three-way ANOVA of N2 peak latencies showed a main effect of Hand (*F*_1,10_ = 7.41, *P* = 0.022). No other significant effects were detected (Table 1). Post hoc two-way ANOVAs (Table 2) failed to detect any effects in either responders or nonresponders that survived correction for multiple comparisons.

#### P2 peak latency.

The three-way ANOVA on the P2 peak latencies did not detect any significant effect. Therefore, post hoc analyses were not performed.

#### ROC curves.

**Fig. 6.**
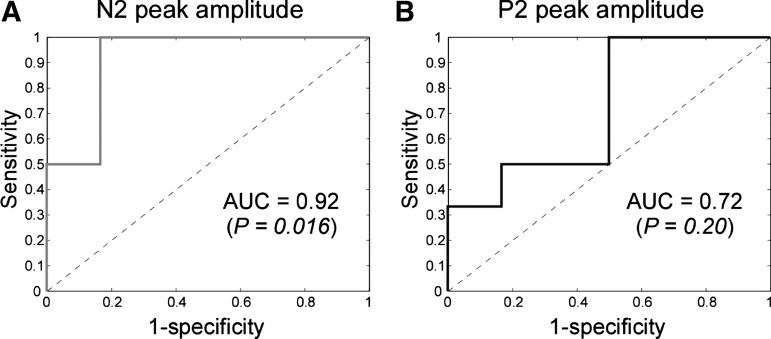
Receiver operating characteristic (ROC) curves and their corresponding AUC values obtained using the interaction term for N2 peak amplitude (*left*) and P2 peak amplitude (*right*) as the predictive factor. Although both measures show predictive ability, only the AUC of N2 ROC was significantly greater than 0.5, indicating that it is therefore a predictor for the state of central sensitization.

The ROC curves obtained from N2 and P2 peak amplitudes are plotted in Fig. 6. The AUC values (±SE) for N2 and P2 were 0.92 ± 0.09 and 0.72 ± 0.16, respectively. Only the AUC for N2 was significantly greater than 0.5 (N2: *P* = 0.016; P2: *P* = 0.200). This suggests that the N2 peak amplitude has adequate sensitivity and specificity for detecting the presence of CS induced by intraepidermal injection of capsaicin.

## DISCUSSION

Developing a biomarker for secondary hyperalgesia, a cardinal symptom of central sensitization (CS), would be useful for both drug discovery and clinical therapy. Such a biomarker would help analgesic drug discovery in early phase trials, facilitate diagnosis of neuropathic pain, and allow objective monitoring of drug treatments in patients.

IES is a technically simple and inexpensive method to selectively stimulate II-AMH skin nociceptors (Inui and Kakigi 2012; Inui et al. 2002; Mouraux et al. 2010). Importantly, IES elicits clear time-locked EEG responses, thus allowing quantification of CS. However, mechanical punctate hyperalgesia is known to be mediated by I-AMH units, rather than II-AMH units (Magerl et al. 2001). Given that IES selectively activates II-AMH units (Mouraux et al. 2010), we tested *1*) whether secondary hyperalgesia from IES coexists with secondary mechanical punctate hyperalgesia and *2*) whether such hyperalgesia is reflected in a corresponding increase in EEG responses.

We obtained several interesting results. First, the intensity of the sensation elicited by IES was significantly increased after intraepidermal injection of capsaicin in those participants who developed robust mechanical punctate hyperalgesia, clearly showing that hyperalgesia from IES occurs and coexists with mechanical hyperalgesia. Second, the peak amplitude of the N2 wave elicited by IES was significantly increased in responders, similarly to the intensity of the sensation elicited by IES. This increased response only occurred when IES were delivered to the hand where capsaicin was injected. Third, ROC analysis showed that the N2 peak amplitude offers the ability to predict the presence of CS with high sensitivity and specificity. These findings suggest that the EEG responses elicited by IES reflect secondary hyperalgesia and thus are a reliable neural correlate of CS.

### Peripheral Afferents Mediating Secondary Hyperalgesia from IES

Although our observations clearly indicate that secondary hyperalgesia elicited by IES coexists with secondary hyperalgesia elicited by mechanical punctate stimuli, it remains unclear whether the two phenomena are mediated by similar populations of Aδ-nociceptors. There is strong physiological evidence that secondary mechanical punctate hyperalgesia is mediated by I-AMH nociceptors. For example, Magerl et al. (2001) demonstrated that secondary mechanical punctate hyperalgesia still occurred in skin that was rendered devoid of II-AMH epidermal terminals by application of high concentrations of topical capsaicin. In contrast, Mouraux et al. (2010) showed that both sensations and EEG responses elicited by IES were abolished in skin that was similarly treated with high-concentration capsaicin, suggesting that IES activates mostly II-AMH nociceptors. It follows that the secondary hyperalgesia from IES observed in this study is likely to be mediated mainly by II-AMH rather than I-AMH nociceptors. However, further experiments are required to confirm whether hyperalgesia from IES and mechanical punctate stimulation are truly mediated by different populations of Aδ-afferents. Nonetheless, it is plausible that, after capsaicin injection, inputs from both I-AMH and II-AMH nociceptors are heterosynaptically facilitated via a common central mechanism and account for the coexistence of secondary hyperalgesia from IES and mechanical punctate stimulation (Ziegler et al. 1999).

### Variability in Capsaicin-Induced Secondary Hyperalgesia

We observed considerable variability in the degree of punctate hyperalgesia that developed after intraepidermal capsaicin injection. Only half of the subjects developed robust hyperalgesia (i.e., a 2-fold increase of pain ratings when the injected hand was stimulated compared with the control hand; Fig. 2).

It is unlikely that this difference between responders and nonresponders was related to the strength of conditioning stimulus, i.e., the activation of C-nociceptors by intraepidermal injection of capsaicin. Indeed, both groups reported similar intensities and durations of burning pain following intraepidermal injection of capsaicin, which suggests that the conditioning stimulus was similar. We note that the development of secondary hyperalgesia can be highly variable even with a highly standardized electrical conditioning stimulus, which suggests considerable differences in the development of CS responses between individuals (Pfau et al. 2011). Furthermore, there is clear evidence that genetic variability contributes to variability in hyperalgesic response following intraepidermal capsaicin injection (Tegeder et al. 2008).

### Brain Potentials Evoked by IES and Central Sensitization: Advantages and Limitations

Previous studies have suggested that brain potentials elicited by punctate mechanical stimulation may be recorded and employed as a potential objective correlates of the CS states (Davies et al. 2010; Iannetti et al. 2013; Kohlloffel et al. 1991). However, as detailed in the Introduction, evoked potentials elicited by punctate mechanical stimuli have significant technical and physiological constrains that hamper clinical translation.

In contrast, IES have several advantages over mechanical punctate stimulation. When delivered at low currents, they are fully selective for Aδ-nociceptors and allow for accurate timing and standardization of stimuli. The stimulating electrode is affordable and can be affixed to any part of the body without difficulty.

The current results show that the amplitude of the ERP elicited by IES of the skin with secondary hyperalgesia clearly reflects that the somatosensory system is centrally sensitized. The amplitude of the N2 wave was significantly larger when IES were delivered to the hand in which capsaicin injection resulted in a clear secondary hyperalgesia (Figs. 4 and 5, Tables 1 and 2). Moreover, the areas under the ROC curves indicate that the change in N2 peak amplitude was significantly predictive of the presence of secondary hyperalgesia (Fig. 6). This result suggests that the changes in N2 amplitude may be developed as a potentially useful biomarker of CS.

Several limitations to IES remain. First, we were unable to isolate the early, contralateral N1 wave typically observed in the brain potentials evoked by nociceptive laser stimuli (Treede et al. 1988; Valentini et al. 2012), most likely because of its lower signal-to-noise ratio. Compared with the subsequent N2-P2 complex, the N1 wave has been shown to better reflect the afferent nociceptive drive (Lee et al. 2009) and appears less susceptible to top-down modulation (for example, placebo manipulation; Martini et al. 2015). These characteristics make the N1 wave a potentially more robust marker for central sensitization. Second, the selective activation of Aδ-nociceptors by IES relies on the use of strictly low-intensity currents. This limitation prevents the recording of stimulus-response functions, because higher intensity currents necessarily entail a coactivation of tactile Aβ-afferents, and therefore a loss of specificity for Aδ-fiber stimulation (Mouraux et al. 2010). Stimulus-response functions are particularly useful for assessing the analgesic potential of novel drugs because they can divulge interactions between stimulus or pain intensity and dose effects. Recording of stimulus-response function using the brain response elicited by mechanical punctate stimuli is similarly problematic because, as detailed earlier, when high forces are exerted, the mechanical punctate stimulus becomes less selective for Aδ-fiber activation (Mouraux et al. 2010; Treede et al. 2002). More recent data reveal that stimulus-response functions can be constructed with the use of IES by varying the number of pulses delivered in quick succession (5-ms intervals) to normal skin; increasing the number of pulses increases the intensity of sensation and EEG amplitudes without changing reaction times or response latencies (Mouraux et al. 2014). Further experiments are required to ascertain if this remains the case after capsaicin-induced hyperalgesia. Moreover, although our present results suggest the potential usefulness of EEG responses to IES as an objective measure of CS, the small sample size used in the present study limits statistical power for detection of smaller effects. Future studies with large samples are needed to confirm the predictive value of IES brain potentials for the state of CS.

### Conclusion

Our study demonstrates that secondary hyperalgesia to IES occurs in a well-recognized experimental model of CS and that the subjective report was corroborated by increased evoked EEG responses. These findings suggest that EEG responses elicited by low-intensity IES, particularly the change in the peak amplitude of the N2 wave, can be used as an objective, physiological correlate of secondary hyperalgesia. Hence, IES evoked potentials hold promise as a low-cost, noninvasive biomarker for CS that can be translated for clinical use with relative ease compared with existing techniques.

## GRANTS

This work was funded by the Wellcome Trust Pain Consortium Award COLL JLARAXR (to G. D. Iannetti and A. H. Dickenson), the European Research Council Consolidator Grant PAINSTRAT (to G. D. Iannetti), a UCL Grand Challenges Studentship (to J. O'Neill), National Natural Science Foundation of China Grant 81571659 (to M. Liang), and Natural Science Foundation of Tianjin Grant 15JCYBJC55100 (to M. Liang).

## DISCLOSURES

No conflicts of interest, financial or otherwise, are declared by the authors.

## AUTHOR CONTRIBUTIONS

M.L., M.C.L., A.H.D., and G.D.I. conception and design of research; M.L., M.C.L., J.O., and G.D.I. performed experiments; M.L., M.C.L., and J.O. analyzed data; M.L., M.C.L., J.O., A.H.D., and G.D.I. interpreted results of experiments; M.L. and G.D.I. prepared figures; M.L., M.C.L., and G.D.I. drafted manuscript; M.L., M.C.L., A.H.D., and G.D.I. edited and revised manuscript; M.L., M.C.L., J.O., A.H.D., and G.D.I. approved final version of manuscript.
